# First Do No Harm With COVID-19: *Corona Collateral Damage Syndrome*

**DOI:** 10.5811/westjem.2020.5.48013

**Published:** 2020-05-22

**Authors:** Lawrence Stock, Mark Brown, Georgienne Bradley

**Affiliations:** * Antelope Valley Hospital, Lancaster, California and David Geffen School of Medicine at UCLA; † Sea Save Foundation, Malibu, California

Communication is complex in that what we say is not always what is heard. Communication that is intended to help can sometimes result in doing harm. The COVID-19 pandemic is a public health emergency. While we rapidly learn of the scientific and healthcare aspects of this disease, there is an opportunity to better understand the consequences of well-intentioned communication by experts.

Given the nature of the rapid global spread of the virus and the high fatality rate of those sick enough to require intensive care, public health and elected-leader messaging of “Stay at Home” was appropriate. With no vaccine or cure, the public health tools of social distancing, respiratory and hand hygiene, and stay-at-home orders were both appropriate and effective at flattening the curve and delaying the peak caseload of COVID-19. Most locations in the US were successful in avoiding overwhelming hospital resources including intensive care units.

However, there are increasing reports from the US and other countries that outside of high-demand hot spots like New York City, most emergency departments (ED) and hospitals have experienced a steep decline in their patient census. ED visits declining 50% or more through the end of April have been widely reported.^1^ Emergency physicians, cardiologists, neurologists, and acute care surgeons wondered, where did all the acute, non-COVID-19 patients go?^2^,^3^ While the number of trauma incidents may have dropped off due to stay-at-home orders, it is unlikely that heart attacks, strokes, and acute surgical emergencies had stopped occurring.

Then we started seeing *delayed presentations* of many diseases with their resulting complications: appendicitis with rupture; completed heart attacks; and strokes with significant deficits, to name a few.^4^,^5^ These are *time-sensitive* conditions in patients who were coming in past the optimal window for treatment. Why did this occur, what role did our messaging play, and how can we correct this in the future?

Corona collateral damage syndrome (CCDS) is the clinical condition resulting from a delay or failure to seek or receive care for acute emergencies for non-COVID-19 medical conditions.^3^ The key cause of CCDS is the fear of catching the virus by coming for care to hospital EDs or other healthcare facilities. This fear appears to have been principally associated with the strong but important message: “Stay at Home.”

This message was said repeatedly by authority figures and amplified by news networks over the past few months. This barrage of messages was effective in getting the public to social distance and stay home. However, the unanticipated collateral damage was the fear of seeking help for *other* concerning symptoms.^6^ We have the opportunity now to course correct and nuance the message:

“If you are having an emergency, go to the Emergency Room. *Hospitals have taken dramatic steps to protect emergency patients from contracting COVID-19.*”

We are reminded that language matters and communication has consequences, some unforeseen. Always best to ask the listener what they heard.
